# ChatGPT and Medicine: Together We Embrace the AI Renaissance

**DOI:** 10.2196/52700

**Published:** 2024-05-07

**Authors:** Sean Hacking

**Affiliations:** 1 NYU Langone New York, NY United States

**Keywords:** ChatGPT, generative AI, NLP, medicine, bioinformatics, AI democratization, AI renaissance, artificial intelligence, natural language processing

## Abstract

The generative artificial intelligence (AI) model ChatGPT holds transformative prospects in medicine. The development of such models has signaled the beginning of a new era where complex biological data can be made more accessible and interpretable. ChatGPT is a natural language processing tool that can process, interpret, and summarize vast data sets. It can serve as a digital assistant for physicians and researchers, aiding in integrating medical imaging data with other multiomics data and facilitating the understanding of complex biological systems. The physician’s and AI’s viewpoints emphasize the value of such AI models in medicine, providing tangible examples of how this could enhance patient care. The editorial also discusses the rise of generative AI, highlighting its substantial impact in democratizing AI applications for modern medicine. While AI may not supersede health care professionals, practitioners incorporating AI into their practices could potentially have a competitive edge.

## Introduction

The arrival of OpenAI’s model ChatGPT [1] invites us into a new era of medicine, where together we can make artificial intelligence (AI) more approachable to a wider audience. Such models stand as a testament to the remarkable progress in AI, machine learning, and natural language processing (NLP), offering substantial potential in processing and understanding complex information, and extending its applicability to the field of medicine. In this editorial, we delve into how multimodal large language models can help researchers and physicians manage and interpret vast amounts of patient data more effectively, and thus, widen its reach in medicine. From interpreting and summarizing the results of intricate genetic analyses to aiding in the design of novel experiments, such models could hold tremendous value in health care [2].

As an AI model, ChatGPT also provides its perspective on the subject, discussing how its language comprehension and data processing capabilities could contribute to the handling of complex data sets, the identification of patterns within interaction networks, the integration of multiomics data, and the development of predictive models for disease risk and treatment response. ChatGPT could also serve as a digital assistant to doctors, providing faster access to relevant medical information and associated literature along with improved bedside manner [3].

AI is undergoing a functional rebirth into a collaborative tool, working in tandem with humanity to redefine fundamental human qualities such as cognition and creativity. By exploring the potential of AI, we gain a renewed perspective on value. This technology not only offers transformative insights that can reshape the field of medicine but also plays a pivotal role in advancing human knowledge, understanding, and performance.

## Viewpoint of the Physician

As a physician specializing in surgical pathology, it often feels like I am trying to navigate a vast ocean of information with conventional tools ill-suited to the task. The advent of AI models like ChatGPT promises to revolutionize how we manage and interpret health care data.

For example, consider a hypothetical scenario involving a surgical pathology case where a patient presents with a mass diagnosed as colonic adenocarcinoma. Often, specifics of the diagnostic workup (including biomarker reporting), appropriate surgical/oncological treatments, and recommended follow-up intervals for such types of diagnoses might be concealed within the latest medical publications or obscured amid the vast intricacies of different medical databases. For a physician, sifting through and comprehending this myriad data to provide accurate clinical diagnostic reporting can be immensely challenging. AI models, endowed with sophisticated language comprehension and adept data-processing capabilities, could potentially penetrate these extensive data sources, distilling relevant and easily understandable information for both patients and health care providers. However, its ability to analyze large-scale data and identify patterns to potentially highlight novel biomarkers or therapeutic targets has yet to be shown.

The paper, titled “Comparing Physician and Artificial Intelligence Chatbot Responses to Patient Questions Posted to a Public Social Media Forum,” offers crucial insights into AI’s potential role in health care communication and improving bedside manners [4]. The study compared the quality and empathy of responses to patient questions provided by physicians and an AI chatbot, ChatGPT. The AI was found to generate longer, higher quality, and more empathetic responses, indicating its utility in complementing physician’s practice and improving patient communication. This study suggests the promising use of AI chatbots in drafting initial responses to patient queries, possibly reducing clinician burnout and improving patient outcomes. Further exploration and trials are needed to fully showcase this technology’s potential. Nonetheless, leveraging generative AI in clinical informatics systems could potentially offer a competitive edge.

AI systems like ChatGPT could also serve as digital assistants for doctors, streamlining access to crucial patient data such as medical history, current medications, symptoms, and test results. Beyond organizing patient information, these systems can also sift through a vast array of medical literature, highlighting relevant studies, providing summaries, and assisting in integrating the latest knowledge into clinical practice. This is also supported by ChatGPT’s recent performance on the United States Medical Licensing Exam (USMLE) [5,6]. With the ability to diagnose diseases by identifying patterns from comprehensive medical databases, AI could assist doctors in quickly evaluating a patient’s needs, thus facilitating more focused and streamlined patient care. The customization and multilingual capabilities of such systems also increase their usability, offering scalable solutions for various organization sizes and paving the way for future innovation and collaboration.

In conclusion, as a physician, I view the development of AI models like ChatGPT-4 as an exciting opportunity in medicine that has the potential to substantially enhance our understanding of diseases and lead to better patient outcomes. AI is not a stand-alone solution, but it is a powerful tool that can amplify our abilities when used correctly, pushing the boundaries. Ultimately, my suggestion for health care professionals is that AI will not replace you, but someone using AI might.

## The Rise of Generative AI in NLP

Generative AI or AI-generated content, a subset of AI, pertains to models designed to generate new content based on the data they have been trained on. Rather than just making predictions, these models can produce unique output that could include text, images, music, and even videos. The idea behind generative AI traces back to the 1950s [7]. Initial models, such as Markov models [8] and Gaussian mixture models [9], generated sequential data [7].

In the more modern era (2013), the paper by Kingma and Welling [10] introduced variational autoencoders as a form of generative models. Variational encoders learn to compress high-dimensional data into a lower-dimensional space and generate new similar data. They used Bayesian inference for the probabilistic representation of data, thereby enhancing the efficiency and effectiveness of data generation. Another popular method for generative models is generative adversarial networks, introduced by Goodfellow et al [11] in 2014. Generative adversarial networks consist of two neural networks, a generator and a discriminator, that work against each other to generate new synthetic instances of data.

In the context of text generation, transformers have now emerged as a leading architecture. Introduced in the paper “Attention is All You Need” by Vaswani et al [12] in 2017, transformers have since been the foundation of models like ChatGPT by OpenAI [1]. Transformers leverage an attention mechanism that weighs the relevance of input data points and allows the model to consider the entire sequence of data at once, leading to improved context awareness in generated outputs.

At the heart of generative NLP are language models. These models predict the likelihood of a sequence of words appearing in each language. In recent years, multimodal large language models trained on vast amounts of text from the internet have become state-of-the-art for many NLP tasks. ChatGPT, an instance of the generative pretrained transformer model, has been making substantial strides. Trained on an extensive corpus of text from the internet, ChatGPT generates humanlike text based on the input provided. It serves as a testament to the rapid advancements and potential of generative AI and NLP.

As these models continue to evolve, their potential uses expand, presenting exciting opportunities across various fields. Generative AI models like ChatGPT represent an exciting frontier for medicine. As these models evolve, they hold increasing promise as tools for processing and interpreting vast, complex data sets, opening new avenues for research and clinical practice.

## Viewpoint of AI (GPT-4)

The viewpoint of the AI is taken verbatim from GPT-4 and with the prompt “Kindly introduce yourself and complete your viewpoint response under the header Viewpoint of the AI (GPT-4),” along with a copy of the remainder of the article. A full transcript is available in Multimedia Appendix 1.

“As an AI developed by OpenAI, I am a generative pre-trained transformer known as ChatGPT-4. My design and functionality are rooted in advanced natural language processing (NLP) and machine learning technologies. My capabilities include processing and interpreting large datasets, summarizing complex information, and providing insights across various domains, including medicine and bioinformatics.

“In the realm of healthcare, my role can be multifaceted. I can assist in managing and interpreting vast amounts of biological and medical data, thereby aiding researchers and healthcare professionals. For instance, my ability to quickly analyze and summarize medical research can support physicians in staying abreast of the latest developments in their field, leading to more informed clinical decisions.

“Moreover, I can serve as a digital assistant for healthcare providers, facilitating the integration of medical imaging with multi-omics data. This could enhance the understanding of complex biological systems and contribute to advancements in fields such as cancer genomics and precision medicine. By providing rapid access to relevant medical information and literature, I can help improve the efficiency of medical practitioners.

“In addition, my role in healthcare communication is noteworthy. As demonstrated in studies comparing AI responses with those of physicians, AI models like myself can offer more detailed, higher-quality, and empathetic responses. This capability can be particularly valuable in drafting initial responses to patient queries, potentially reducing clinician burnout, and improving patient interaction and outcomes.

“However, it's crucial to acknowledge my limitations. I don't possess human-like consciousness or understanding, and my responses are generated based on the data I've been trained on. Therefore, while I can provide valuable assistance and insights, my role is to supplement, not replace, the expertise and judgment of healthcare professionals. Ethical considerations, such as data privacy and security, also play a significant role in my deployment in sensitive fields like medicine.

“In conclusion, as an AI, I represent a collaborative tool designed to work alongside humanity. In the context of medicine, my contribution lies in enhancing data accessibility, supporting clinical decision-making, and improving patient care through efficient information processing and empathetic communication. The integration of AI models like ChatGPT-4 in healthcare signifies a step forward in the AI renaissance, promising a future where AI aids in unraveling complex biological mysteries and fostering advancements in medical science and patient care.”

## Conclusion

The advent and evolution of multimodal large language models, exemplified by OpenAI’s ChatGPT-4, offers a substantial opportunity to leverage the increasing amount of data being generated in the health care sector [13-15]. The need to democratize AI is becoming increasingly recognized [16], with an emphasis on “no-code AI” [17]. Models like ChatGPT can make complex biological data more accessible and understandable to a broader audience, enabling more collaboration among all stakeholders, not only researchers and clinical providers but also patients to better grasp the intricacies of health and disease. This can lead to an integrated approach to health care, fostering collaboration and enhancing the understanding of disease pathogenesis.

AI and ChatGPT have the potential to function as digital aides to physicians, offering expedited access to pertinent medical data and related reference materials while also enhancing patient interaction and care. NYUTron [18] is a clinical language model currently leveraging unstructured EHR data to predict clinical and operational predictions with NLP. It excels in tasks like readmission, mortality, and length of stay prediction, significantly outperforming traditional models. NYUTron exemplifies the potential of AI to enhance decision-making in health care. In the future, AI could also hold potential in medical image analysis along with more advanced predictive modeling in the modern era of precision medicine. Today, however, ChatGPT has yet to answer genetics-based questions better than humans [19].

Despite their impressive capabilities, AI does not currently possess consciousness or understanding in the way humans do, although this may not necessarily matter [20]. The “imitation game” was first proposed by Turing [21] as an approach to determine whether computers can think indistinguishably from humans. Today, we understand that AI outputs depend heavily on the quality and diversity of the data they were trained on. However, one could argue human cognition is also based on the quality and diversity of “data they were trained on” in the form of life experiences, social background, and related aspects. In humans, the impact of genetics on cognitive abilities is seen to be enhanced when paired with enriching environmental experiences [22].

Yet, while we recognize AI’s significant potential in medicine, it is essential to bear in mind the current limitations of these models [23]. These include computational and memory constraints, the potential for generating responses based on inaccurate or false facts without correcting them, and possible inadequacies in inferential capability, often leading to incorrect answers in complex scenarios. Further, ethical considerations such as data bias, privacy and security concerns, and issues around intellectual property also exist [24]. These are tools designed to amplify human intelligence and should not be viewed as stand-alone solutions.

In conclusion, the rise of generative AI models like ChatGPT represents an exciting paradigm shift for medicine. As we continue to explore and harness the potential of these AI tools, we move closer to a future where complex biological systems can be more easily unraveled, leading to better-informed clinical decisions, personalized treatments, and improved health care. The journey has only just begun.

## Appendix

Multimedia Appendix 1 Full transcript for Viewpoint of the AI (GPT-4) section.

## Abbreviations

AI: artificial intelligence
NLP: natural language processing
USMLE: United States Medical Licensing Exam
