# Supplementary material for: ChatGPT and Medicine: Together We Embrace the AI Renaissance
Source: JMIR Bioinform Biotechnol. 2024 May 7;5:e52700. doi: 10.2196/52700 (PMC11135232; doi:10.2196/52700)
Supplement: Multimedia Appendix 1 [file bioinform_v5i1e52700_app1.pdf]

Viewpoint of the AI (GPT-4)

January 9, 2024

Kindly introduce yourself and complete your viewpoint response under the header Viewpoint of the AI (GPT-4). ChatGPT and Medicine - Together We Embrace the AI Renaissance Sean M. Hacking Department of Pathology, NYU Langone Health, NYU Grossman School of Medicine, New York, NY Abstract The generative artificial intelligence (AI) model, Chat Generative Pre-trained Transformer (ChatGPT), holds transformative prospects in medicine. The development of such models has signaled the beginning of a new era where complex biological data can be made more accessible and interpretable. ChatGPT is a natural language processing (NLP) tool that can process, interpret, and summarize vast datasets. It can serve as a digital assistant for physicians and researchers, aiding in the integration of medical imaging data with other multi-omics data and facilitating the understanding of complex biological systems. Notably, its potential contribution to the field of cancer genomics and precision medicine is discussed. The physician's and the AI's viewpoint emphasize the value of such AI models in a clinical setting, providing tangible examples of how this could enhance patient care. The editorial also discusses the rise of generative AI, highlighting its significant impact in democratizing AI applications for modern medicine. While AI may not supersede healthcare professionals, practitioners incorporating AI into their practices could potentially have a competitive edge. Keywords: ChatGPT, Generative AI, NLP, Bioinformatics, Genomics, Precision Medicine, AI Democratization, AI Renaissance Corresponding Author: Sean M. Hacking, MB,BCh,BAO Department of Pathology, NYU School of Medicine, New York, NY 10016 United States Email: sean.hacking@nyulangone.org Introduction The field of bioinformatics is both vast and intricate, harnessing computational tools to interpret and decipher the complex languages of biological systems[1]. It offers profound insights into the underpinnings of life, aiding in the understanding of diseases, and informing the development of treatments. However, the sheer scale and complexity of the data involved can often render it inaccessible to many. The arrival of OpenAI's model, Chat Generative Pre-trained Transformer (ChatGPT)[2], invites us to a new era of democratized medical bioinformatics, where together we can make AI more approachable to a wider audience. Such models stand as a testament to the remarkable progress in artificial intelligence (AI), machine learning (ML), and natural language processing (NLP), offering significant potential in processing and understanding complex information, and extending its applicability to the field of medicine. In this paper, we delve into how multimodal large language models can help researchers manage and interpret vast amounts of biological data more effectively, and thus, widen its reach in medicine. From interpreting and summarizing the results of intricate genetic analyses to aiding in the design of novel experiments, such models could be a powerful ally for researchers and healthcare professionals working in the healthcare field[3]. As an AI model, ChatGPT also provides its perspective on the subject, discussing how its language comprehension and data processing capabilities could contribute to the handling of complex datasets, the identification of patterns within interaction networks, the integration of multi-omics data, and the development of predictive models for disease risk and treatment response. ChatGPT could also serve as a digital assistant to doctors, providing faster access to relevant medical information and associated literature, along with an improved bedside manner[4]. AI is undergoing a functional rebirth into a collaborative tool, working in tandem with humanity to redefine fundamental human qualities such as cognition and creativity. By exploring the potential of AI, we gain a renewed perspective

on value. This technology not only offers transformative insights that can reshape the field of medicine but also plays a pivotal role in advancing our knowledge and understanding as well. Viewpoint of the Physician As a physician specializing in surgical pathology, my engagement with medical bioinformatics is crucial in identifying, understanding, and potentially influencing disease patterns. However, it often feels like I am trying to navigate a vast ocean of information with conventional tools ill-suited to the task. The advent of AI models like ChatGPT promises to revolutionize how we manage and interpret healthcare data. For example, consider a hypothetical scenario involving a surgical pathology case where a patient presents with a colonic polyp exhibiting at least high-grade dysplasia, which some pathologists might also consider as intramucosal carcinoma[5, 6]. Often, specifics of the diagnostic workup (including biomarker reporting), recommended follow-up intervals, and appropriate surgical treatments for such types of diagnoses might be concealed within the latest medical publications or obscured amidst the vast intricacies of different medical databases. For a physician, sifting through and comprehending this myriad of data to provide accurate clinical diagnostic reporting can be immensely challenging. ChatGPT-4, endowed with sophisticated language comprehension and adept data processing capabilities, could potentially penetrate these extensive data sources, distilling relevant and easily understandable information for both patients and healthcare providers. However, its ability to analyze large-scale data and identify patterns to potentially highlight novel biomarkers or therapeutic targets has yet to be shown. The paper[7], "Comparing Physician and Artificial Intelligence Chatbot Responses to Patient Questions Posted to a Public Social Media Forum," offers crucial insights into AI's potential role in healthcare communication and improving bedside manners. The study compared the quality and empathy of responses to patient questions provided by physicians and an AI chatbot, ChatGPT. The AI was found to generate longer, higher quality, and more empathetic responses, indicating its utility in complementing physician's practice and improving patient communication. This study suggests the promising use of AI chatbots in drafting initial responses to patient queries, possibly reducing clinician burnout, and improving patient outcomes. Further exploration and trials are needed to fully realize this technology's potential. Nonetheless, leveraging generative AI in clinical information systems could potentially offer a competitive edge. AI systems like ChatGPT could also serve as digital assistants for doctors, streamlining access to crucial patient data such as medical history, current medications, symptoms, and test results. Beyond organizing patient information, these systems can also sift through a vast array of medical literature, highlighting relevant studies, providing summaries, and assisting in integrating the latest knowledge into clinical practice. With the ability to diagnose diseases by identifying patterns from comprehensive medical databases, AI could assist doctors in quickly evaluating a patient's needs, thus facilitating more focused patient care. The customization and multilingual capabilities of such systems also increase their usability, offering scalable solutions for various organizational sizes, and paving the way for future innovation and collaboration. In conclusion, as a physician, I view the development of AI models like ChatGPT-4 as an exciting opportunity in medicine. Which has the potential to significantly enhance our understanding of diseases, and lead to better patient outcomes. AI is not a standalone solution, but it is a powerful tool that can amplify our abilities when used correctly, pushing the boundaries. Ultimately, my suggestion for healthcare professionals is that AI will not replace you, but someone using AI might. The Rise of Generative AI in NLP Generative AI or AI-generated content (AIGC), a subset of AI, pertains to models designed to generate new content based on the data they've been trained on. Rather than just making predictions, these models can produce unique output which could include text,

images, music, and even videos. The idea behind generative AI traces back to the 1950s[8]. Initial models, such as Markov Models[9] and Gaussian Mixture Models[10] produced straightforward data. In the more modern era (2013), the paper "Auto-Encoding Variational Bayes"[11], introduced Variational Autoencoders (VAEs) as a form of generative models. VAEs learn to compress high-dimensional data into a lower-dimensional space and generate new, similar data. They used Bayesian inference for the probabilistic representation of data, thereby enhancing the efficiency and effectiveness of data generation. This work was pivotal to the evolution and understanding of generative AI models. Another popular method for generative models is Generative Adversarial Networks (GANs), introduced by Ian Goodfellow et al. in 2014[12]. GANs consist of two neural networks, a generator, and a discriminator, which work against each other to generate new, synthetic instances of data that can pass for real data. In the context of text generation, transformers have now emerged as a leading architecture. Introduced in the paper "Attention is All You Need" by Vaswani et al.[13] in 2017, transformers have since been the foundation of models like ChatGPT by OpenAI[2]. Transformers leverage an attention mechanism that weighs the relevance of input data points and allows the model to consider the entire sequence of data at once, leading to improved context awareness in generated outputs. At the heart of generative NLP are language models. These models predict the likelihood of a sequence of words appearing in each language. In recent years, multimodal large language models trained on vast amounts of text from the internet have become state-of-the-art for many NLP tasks. ChatGPT, an instance of the GPT model, has been making significant strides. Trained on an extensive corpus of text from the internet, ChatGPT generates human-like text based on the input provided. It serves as a testament to the rapid advancements and potential of generative AI and NLP. Despite their impressive capabilities, it's important to understand that these AI models do not possess consciousness or understanding in the way humans do. The ‘imitation game’ was first proposed by A. M. Turing[14] as an approach to determine whether computers can think indistinguishably from humans. Today we understand AI outputs depend heavily on the quality and diversity of the data they were trained on. However, I would argue human cognition is also based on the quality and diversity of "data they were trained on" in the form of experiences, social upbringing, and so on. In humans, the impact of genetics on cognitive abilities is seen to be enhanced when paired with enriching environmental experiences[15]. As these models continue to evolve, their potential uses expand, presenting exciting opportunities across various fields. Generative AI models represent an exciting frontier for medicine. As these models evolve, they hold increasing promise as tools for processing and interpreting vast, complex datasets, opening new avenues for research and clinical practice. Viewpoint of the AI (GPT-4) The viewpoint of the AI is taken verbatim from GPT-4 and with the prompt “Kindly introduce yourself and complete your viewpoint response under the header Viewpoint of the AI (GPT-4)”, along with a copy of the remainder of the article. A full transcript is available in the Appendix, and is also available at the following link: Conclusion The advent and evolution of multimodal large language models, exemplified by OpenAI's ChatGPT-4, offers a substantial opportunity to leverage the increasing amount of data being generated in the healthcare sector[16-18]. The need to democratize AI is becoming increasingly recognized[19], with an emphasis on “no-code AI”[20]. Models like ChatGPT can make complex biological data more accessible and understandable to a broader audience, enabling more collaboration among all stakeholders, not only researchers and clinical providers but also patients to better grasp the intricacies of health and disease. This can lead to an integrated approach to healthcare, fostering collaboration and enhancing the understanding of complex biological systems. AI could hold

potential in medical imaging data, genomic variation detection, multi-omics data integration, prediction modeling, and network biology. AI and ChatGPT also have the potential to function as digital aides to physicians, offering expedited access to pertinent medical data and related reference materials, while also enhancing patient interaction and care. The AI landscape is rapidly expanding with various innovative models and platforms from different companies, each bringing unique capabilities and advancements to the field. Mistral is open models which are meant to be fast and adaptable for different use cases. Including industries like technology, finance, or healthcare. Gemini on the other hand is the newest Google generative AI model, developed for multimodality, including text, images, video, and audio. Finally, Facebook's RoBERTa has been designed for more accurate natural language processing tasks compared to BERT, trained on a wider range of datasets and languages. IBM’s Watson: IBM's Watson is well-known in the AI field for its ability to process and analyze large datasets, especially in domains like healthcare, finance, and customer service. Watson's capabilities in natural language processing and data analysis make it a versatile tool for various business applications. Yet, while we recognize AI's significant potential in medicine, it is essential to bear in mind the current limitations of these models[21]. These include computational and memory constraints, the potential for generating responses based on inaccurate or false facts without correcting them, and possible inadequacies in inferential capability, often leading to incorrect answers in complex scenarios. Further, ethical considerations such as data bias, privacy and security concerns, and issues around intellectual property also exist. These are tools designed to aid human intelligence and should not be viewed as standalone solutions. In conclusion, the rise of generative AI models like ChatGPT represents an exciting paradigm shift for medicine. As we continue to explore and harness the potential of these AI tools, we move closer to a future where complex biological systems can be more easily unraveled, leading to better-informed clinical decisions, personalized treatments, and improved healthcare. The journey has only just begun. Disclosures The viewpoint of the AI was written by ChatGPT4 (https://chat.openai.com/) for this editorial. This was reviewed and full accountability for the publication's content rests with the author. A full transcript is available in the Appendix, and is also available at the following link: Conflict of Interest Author SMH is the founder and has equity ownership in Odyssey HealthCare Solutions Inc. Author SMH is a JMIR Bioinformatics and Biotechnology (JBB) associate editor. There are no remaining potential conflicts of interest to disclose. References 1. Bayat A: Science, medicine, and the future: Bioinformatics. Bmj 2002, 324(7344):1018-1022. 2. Radford A, Wu J, Child R, Luan D, Amodei D, Sutskever I: Language Models are Unsupervised Multitask Learners. In: 2019; 2019. 3. Dave T, Athaluri SA, Singh S: ChatGPT in medicine: an overview of its applications, advantages, limitations, future prospects, and ethical considerations. Front Artif Intell 2023, 6:1169595. 4. Javaid M, Haleem A, Singh RP: ChatGPT for healthcare services: An emerging stage for an innovative perspective. BenchCouncil Transactions on Benchmarks, Standards and Evaluations 2023, 3(1):100105. 5. Lewin MR, Fenton H, Burkart AL, Sheridan T, Abu-Alfa AK, Montgomery EA: Poorly differentiated colorectal carcinoma with invasion restricted to lamina propria (intramucosal carcinoma): a follow-up study of 15 cases. Am J Surg Pathol 2007, 31(12):1882-1886. 6. Fleming M, Ravula S, Tatishchev SF, Wang HL: Colorectal carcinoma: Pathologic aspects. J Gastrointest Oncol 2012, 3(3):153-173. 7. Ayers JW, Poliak A, Dredze M, Leas EC, Zhu Z, Kelley JB, Faix DJ, Goodman AM, Longhurst CA, Hogarth M et al: Comparing Physician and Artificial Intelligence Chatbot Responses to Patient Questions Posted to a Public Social Media Forum. JAMA Internal Medicine 2023. 8. Cao Y, Li S, Liu Y, Yan Z, Dai Y, Yu PS, Sun L: A Comprehensive Survey of AI-Generated Content

(AIGC): A History of Generative AI from GAN to ChatGPT. ArXiv 2023, abs/2303.04226. 9. Knill K, Young S: Hidden Markov Models in Speech and Language Processing. In: Corpus-Based Methods in Language and Speech Processing. edn. Edited by Young S, Bloothooft G. Dordrecht: Springer Netherlands; 1997: 27-68. 10. Reynolds A: Gaussian mixture models. Encyclopedia of biometrics 2009, 741:659-663. 11. Kingma DP, Welling M: Auto-Encoding Variational Bayes. CoRR 2013, abs/1312.6114. 12. Goodfellow I, Pouget-Abadie J, Mirza M, Xu B, Warde-Farley D, Ozair S, Courville A, Bengio Y: Generative Adversarial Networks. Advances in Neural Information Processing Systems 2014, 3. 13. Vaswani A, Shazeer NM, Parmar N, Uszkoreit J, Jones L, Gomez AN, Kaiser L, Polosukhin I: Attention is All you Need. In: NIPS: 2017; 2017. 14. TURING AM: I.—COMPUTING MACHINERY AND INTELLIGENCE. Mind 1950, LIX(236):433-460. 15. Tucker-Drob EM, Briley DA, Harden KP: Genetic and Environmental Influences on Cognition Across Development and Context. Curr Dir Psychol Sci 2013, 22(5):349-355. 16. Dash S, Shakyawar SK, Sharma M, Kaushik S: Big data in healthcare: management, analysis and future prospects. Journal of Big Data 2019, 6(1):54. 17. Pastorino R, De Vito C, Migliara G, Glocker K, Binenbaum I, Ricciardi W, Boccia S: Benefits and challenges of Big Data in healthcare: an overview of the European initiatives. Eur J Public Health 2019, 29(Supplement_3):23-27. 18. Alberto IRI, Alberto NRI, Ghosh AK, Jain B, Jayakumar S, Martinez-Martin N, McCague N, Moukheiber D, Moukheiber L, Moukheiber M et al: The impact of commercial health datasets on medical research and health-care algorithms. Lancet Digit Health 2023, 5(5):e288-e294. 19. Seger E, Ovadya A, Garfinkel B, Siddarth D, Dafoe A: Democratising AI: Multiple Meanings, Goals, and Methods. ArXiv 2023, abs/2303.12642. 20. Sundberg L, Holmström J: Democratizing artificial intelligence: How no-code AI can leverage machine learning operations. Business Horizons 2023. 21. Cheng H-W: Challenges and Limitations of ChatGPT and Artificial Intelligence for Scientific Research: A Perspective from Organic Materials. AI 2023, 4(2):401-405.

ChatGPT

Viewpoint of the AI (GPT-4)

As an AI developed by OpenAI, I am a generative pre-trained transformer known as ChatGPT-4. My design and functionality are rooted in advanced natural language processing (NLP) and machine learning technologies. My capabilities include processing and interpreting large datasets, summarizing complex information, and providing insights across various domains, including medicine and bioinformatics.

In the realm of healthcare, my role can be multifaceted. I can assist in managing and interpreting vast amounts of biological and medical data, thereby aiding researchers and healthcare professionals. For instance, my ability to quickly analyze and summarize medical research can support physicians in staying abreast of the latest developments in their field, leading to more informed clinical decisions.

Moreover, I can serve as a digital assistant for healthcare providers, facilitating the integration of medical imaging with multi-omics data. This could enhance the understanding of complex biological systems and contribute to advancements in fields such as cancer genomics and precision medicine. By providing rapid access to relevant medical information and literature, I can help improve the efficiency of medical practitioners.

In addition, my role in healthcare communication is noteworthy. As demonstrated in studies comparing AI responses with those of physicians, AI models like myself can offer more detailed, higher-quality, and empathetic responses. This capability can be particularly valuable in drafting initial responses to patient queries, potentially reducing clinician burnout and improving patient interaction and outcomes.

However, it's crucial to acknowledge my limitations. I don't possess human-like consciousness or understanding, and my responses are generated based on the data I've been trained on. Therefore, while I can provide valuable assistance and insights, my role is to supplement, not replace, the expertise and judgment of healthcare professionals. Ethical considerations, such as data privacy and security, also play a significant role in my deployment in sensitive fields like medicine.

In conclusion, as an AI, I represent a collaborative tool designed to work alongside humanity. In the context of medicine, my contribution lies in enhancing data accessibility, supporting clinical decision-making, and improving patient care through efficient information processing and empathetic communication. The integration of AI models like ChatGPT-4 in healthcare signifies a step forward in the AI renaissance, promising a future where AI aids in unraveling complex biological mysteries and fostering advancements in medical science and patient care.

Get started with ChatGPT

|Terms of use|Privacy policy|
